# Timing and cause of perinatal mortality for small-for-gestational-age babies in South Africa: critical periods and challenges with detection

**DOI:** 10.1186/s40748-016-0039-4

**Published:** 2016-10-21

**Authors:** Tina Lavin, David B. Preen, Robert Pattinson

**Affiliations:** 1 Centre for Health Services Research, School of Population Health, The University of Western Australia (M431), 35 Stirling Highway, Crawley, WA 6009 Australia; 2 SAMRC Maternal and Infant Health Care Strategies, Department of Obstetrics and Gynaecology, University of Pretoria, Pretoria, South Africa

**Keywords:** Small-for-gestational-age, Intrauterine growth restriction, Perinatal mortality, South Africa, Low-and-middle-income countries, Doppler

## Abstract

**Background:**

Little information exists on timing and cause of death for small-for-gestational-age (SGA) babies in low-and-middle-income-countries (LMICs), despite evidence from high-income countries suggesting critical periods for SGA babies. This study explored the timing and cause of stillbirth and early neonatal mortality (END, <7 days) by small-for-gestational age in three provinces in South Africa. In South Africa, the largest category of perinatal deaths is unexplained stillbirth, of which up to one-quarter have intra-uterine growth restriction.

**Methods:**

Secondary analysis of the South African Perinatal Problems Identification Program (PPIP) database allowed for the analysis of gestational age at death and clinically confirmed diagnosis of stillbirth and early neonatal death (END) (>1000 g and >28 weeks) across gestation. Comparisons by province, size-for-gestational-age, gestational age groups, and maternal condition at death were performed. The provinces investigated were: Western Cape (fortnightly antenatal care visits from 32 to 38 weeks), Limpopo and Mpumalanga (no antenatal care visits between 32 to 38 weeks).

**Results:**

There were 528,727 births in the study period and 8111 stillbirths and 5792 early neonatal deaths. Similar timing of deaths across gestation was seen for the three provinces with the greatest proportion of deaths for SGA babies at 33–37 weeks (stillbirths 52.9 %; END 43.3 %; *p* < 0.05). SGA babies had a greater proportion of deaths due to hypertension (SGA22.9 %; AGA 18.6 %; LGA 18.6 %; *p* < 0.05) and intrauterine growth restriction (SGA 6.8 %; AGA 1.7 %; LGA 1.4 %; *p* < 0.05). No increase was seen in poor maternal condition for SGA babies and 54.9 % of deaths had a healthy mother. Of mothers that were healthy the greatest proportion of SGA stillbirths were due to unexplained intrauterine death (53.9 %).

**Conclusion:**

There was a peak in stillbirths for SGA babies 33–37 weeks in all provinces. Detecting SGA is further complicated as in most cases the mother is healthy. Further research into Umbiflow Doppler velocimetry use in low-risk populations is warranted and may be a viable strategy to increase current detection of SGA babies at risk of mortality in LMICs.

## Background

Approximately 25 % of children born in low-and-middle-income countries (LMICs) are small-for-gestational-age (SGA) [1]. Babies who are SGA are at increased risk of mortality and neonatal morbidity [2, 3], making the detection and clinical management of such infants crucial. The condition may constitute a small but healthy fetus or be due to pathological growth failure (intrauterine growth restriction (IUGR)) [4]. The purpose of identifying SGA fetuses, defined as a birth weight in the lowest decile on standard growth curves [5], is to recognise those most at risk of poor outcomes [3].

Studies to date have indicated that there may be a critical period for increased mortality for SGA babies [3, 5, 6]. A UK study found that stillbirths between 28 and 36 weeks were increased for SGA babies [5]. Other studies have found that SGA babies are at increased risk of stillbirth compared to non-SGA babies at all gestational ages [3], and that the risk of stillbirth for SGA babies increases with advancing gestational age [6].

In South Africa, the largest category of perinatal deaths is unexplained stillbirth, of which up to one-quarter have IUGR [7]. Early recognition can prevent some of these deaths. Early antenatal detection of SGA babies remains a challenge in LMICs, but is important as most deaths occur in the late preterm or term period, where survival of live born infants in well-resourced units is high [8]. As detection of SGA at the population level is challenging due to resource constraints, identifying critical periods and causes of mortality across gestation may elucidate the best approach.

This study explores the gestational age at death and cause of stillbirth and early neonatal mortality (up to 7 days neonatal life) by size-for-gestational age in three South African provinces.

## Methods

Secondary analysis of the South African Perinatal Problems Identification Program (PPIP) database allowed for the analysis of gestational age at death and clinically confirmed diagnosis of stillbirth and early neonatal death (END) across gestation. The program also allowed for comparisons between SGA, appropriate-for-gestational-age (AGA) and large-for-gestational-age (LGA) babies from 1 October and 2013 and 31 August 2015 and between three provinces: Western Cape, Limpopo and Mpumalanga. Western Cape has fortnightly antenatal care visits between 32 and 38 weeks, while Limpopo and Mpumalanga do not have visits between 32 and 38 weeks, preventing the opportunity for detection of SGA at these gestations. These provinces were chosen (from nine available) as they have the greatest PPIP coverage, auditing >90 % of perinatal deaths. PPIP is a perinatal quality audit system that has been described in detail elsewhere [7, 9]. Briefly, at each clinical site (*n* = 292) across the three provinces the clinical team perform a review shortly after a death has occurred. The primary obstetric cause of death was defined by the PPIP technical team as the main obstetric event or pregnancy occurrence which was integral in the pathway to perinatal death, as described in other published studies [7]. IUGR was also identified at the time of death through clinical evaluation. Maternal condition at the time of death was also recorded. Categories for the primary obstetric cause of stillbirth and early neonatal death were as follows: antepartum haemorrhage, spontaneous preterm labour (intrapartum stillbirth), unexplained intrauterine death, fetal abnormality, hypertensive disorders, infections, intrapartum asphyxia, intrauterine growth restriction, maternal disease, miscellaneous (rhesus isoimmunisation, twin-to-twin transfusion, extra-uterine pregnancy and other cause of death not described), no obstetric cause and trauma. The maternal condition is defined as either healthy (where the clinician examining her could not find any clinical problems) or the occurrence of a recognised medical or obstetric complication (eg. cardiac, endocrine, respiratory disease or other disease that are an indirect cause of morbidity), categorised as coincidental conditions, medical and surgical disorders, non-pregnancy related infections, extra-uterine pregnancy, pregnancy-related sepsis, obstetric haemorrhage, hypertension, anaesthetic complications, embolism, and acute collapse (cause unknown).

Gestational age was calculated based on date of last menstrual period, ultrasound or clinical examination and cases were excluded if the gestation age was unknown or if the estimated age was considered ‘uncertain’. There was no hierarchy employed based on method of gestational age estimation. Detailed data were extracted on all stillbirths >1000 g and 28–42 weeks gestation and early neonatal deaths less than 7 days of neonatal life. Data were only included up to 42 weeks gestation as Theron weight distribution curves are not considered reliable for growth measurements after 42 weeks gestation [10, 11]. Only women who had reported receiving antenatal care were included as SGA can be detected antenatally. Data extracted from the three provinces represent 58.6 % of all deaths in South Africa that met the study criteria. Birth weight for gestation was obtained from Theron charts. SGA was defined as neonates with <10th centile for gestational age based on South African specific growth charts [10, 11].

### Statistical analysis

Stillbirth and early neonatal death cumulative incidence were calculated for each province using the number of reported births (all births for stillbirth, live births for neonatal death rate) as the denominator. The frequencies of deaths occurring across gestation were compared between SGA, AGA and LGA babies as well as between provinces. Comparisons between the proportion of primary cause of deaths were made by size-for-gestational-age, gestational age at death and in relation to maternal conditions. Gestational age was grouped into three categories for analysis: 28–32 weeks, 33–37 weeks and 38–42 weeks. Frequency distributions were performed and Pearson’s chi-squared test or Fisher’s exact test (where *n* < 5) were used to determine crude differences between proportions for the key comparisons made (i.e. size-for-gestational-age, gestational age at death, maternal condition and between provinces). Independent t-tests were used to compare means between key groups. A *p*-value <0.05 was accepted as statically significant.

The PPIP program has ethical approval from the Faculty of Heath Sciences Ethics Committee at the University of Pretoria. The data are collected with permission from the South African Department of Health. This secondary analysis was approved by the PPIP technical task team and UWA Human Ethics Committee.

## Results

There were 528,727 births >1000 g in the study period (Mpumalanga = 145,362; Western Cape = 173,597; Limpopo = 209,768), of which 8111 (1.5 %) were stillbirths (Mpumalanga =2501; Limpopo = 3808; Western Cape = 1802) and 3792 (0.7 %) died in the early neonatal period (<7 days) (Mpumalanga =1163; Limpopo = 2124; Western Cape = 505). The cumulative incidence of stillbirth for the study period was highest in Limpopo (18.2 per 1000 births) and Mpumalanga (17.2/1000) compared to Western Cape (10.4/1000). The cumulative incidence of early neonatal death was highest in Limpopo at 10.3 per 1000 live births, followed by Mpumalanga (8.1 per 1000) and Western Cape (3.0 per 1000). After exclusion of deaths prior to 28 weeks (and after 42 weeks), deaths with unknown or uncertain gestation and women who had not received ANC, the number of deaths used for analysis was 6133 (Mpumalanga *n* = 2198; Limpopo *n* = 3032; Western Cape *n* = 903). The greatest proportion of babies in the study were AGA (61.5 %), followed by SGA (22.6 %) and LGA (12.3 %).

### Gestational age at death

**Fig. 1:**
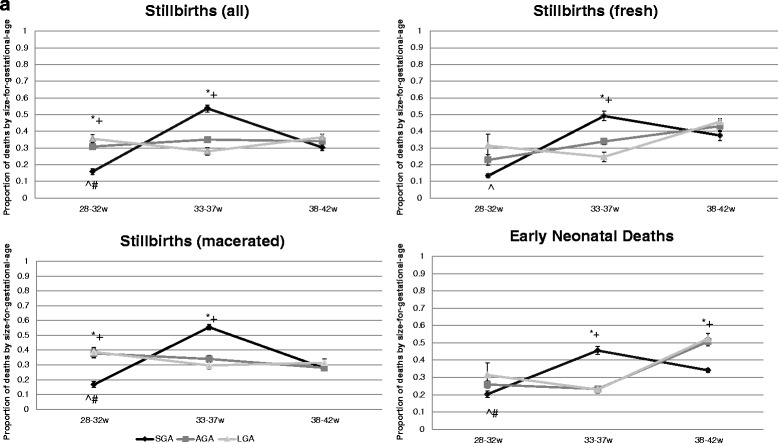
Proportion of deaths (standard error for provinces combined) across gestation by size-for-gestational-age. *p < 0.05 with AGA; +*p* < 0.05 with LGA. ^SGA significantly increased at 33–37w compared to 28–32w; #SGA significantly increased at 33–37w compared to 38–42 weeks. Legend: SGA (*black line*); AGA (*gray line*); LGA (*light gray line*)

There were no differences between provinces in terms of representation and timing of stillbirths between SGA, AGA, LGA, therefore data from the three provinces were combined and presented as means and standard errors. Stillbirths who were AGA or LGA occurred across gestation without any significant increases or decreases. A larger proportion of SBs occurred for SGA babies in the 33–37 week period in all provinces (Fig. 1). When considering macerated and fresh stillbirths a peak at 33–37 weeks was also seen for SGA babies (Fig. 1). A similar pattern was seen for early neonatal deaths (END) with a peak at 33–37 weeks for SGA babies. The timing of END for AGA and LGA babies increased during the 38–42 week period but remained low at 28–32w and 33–37w.

### Primary causes of death

#### Stillbirths by size-for-gestational-age

The primary cause of stillbirth across the sample was unexplained intrauterine death (33.1 %), followed by intrapartum asphyxia (17.2 %) and hypertensive disorders (19.7 %) (Table 1). Some primary causes of death were more frequent in the SGA group, such as fetal abnormality (37.8 %) and intrauterine growth restriction (59.8 %) which represented up to half of all deaths despite only representing 26.2 % of the total sample (*p* < 0.05). Intrapartum asphyxia for SGA babies was indicated in 13.3 % of deaths due to intrapartum asphyxia across gestation (*p* < 0.05).

**Table 1:** Primary cause of stillbirth by size-for-gestation (Western Cape, Limpopo and Mpumalanga combined); *n* = 4059

Primary cause of stillbirth	SGA	AGA	LGA	
	*n* (%)	*n* (%)	*n* (%)	All %
APH	145 (13.6)	399 (16.0)	51 (10.2)	14.7
Unexplained Intrauterine death	367 (34.5)	812 (32.5)	164 (32.8)	33.1
Fetal abnormality	54 (5.1)*	67 (2.7)	22 (4.4)	3.5
Hypertensive disorders	244 (22.9)*^	463 (18.6)	93 (18.6)	19.7
Infections	26 (2.4)	42 (1.7)	7 (1.4)	1.8
Intrapartum asphyxia	93 (8.7)*^	512 (20.5)	93 (18.6)	17.2
Intrauterine growth restriction	73 (6.8)*^	42 (1.7)	7 (1.4)	3.0
Maternal disease	12 (1.1)*^	56 (2.2)	41 (8.2)	2.7
Misc.	22 (2.1)	27 (1.1)	6 (1.2)	1.4
No obstetric cause	1 (0.1)	2 (0.1)	1 (0.2)	0.1
Spont. Preterm labour	27 (2.5)	65 (2.6)	12 (2.4)	2.6
Trauma	1 (0.1)	8 (0.3)	2 (0.4)	0.3
All	1065	2495	499	100.0

*p < 0.05 with AGA; ^*p* < 0.05 with LGA; Pearson chi-sq tests, Fisher’s exact where cell count <5

#### Early neonatal deaths by size-for-gestational-age

The primary cause of early neonatal death (<7 days) was intrapartum asphyxia (42.1 %), followed by spontaneous preterm labour (18.7 %), fetal abnormality (11.4 %) and hypertensive disorders (7.3 %) (Table 2). Cause of death in SGA babies were intrauterine growth restriction (91.3 %), hypertensive disorders (37.0 %), fetal abnormality (33.3 %), and spontaneous preterm labour (36.0 %) which represented a large proportion of all deaths by cause despite representing only 23.7 % of the sample.

**Table 2:** Primary cause of early neonatal death (<7 days) by size-for-gestational age (provinces combined); *n* = 1742

Primary cause death (END)	SGA	AGA	LGA	
	*n* (%)	*n* (%)	*n* (%)	All %
APH	18 (44)	59 (5.1)	3 (1.6)	4.6
Unexplained Intrauterine death	1 (0.2)	2 (0.2)	0 (0.0)	0.2
Fetal abnormality	66 (16.0)*	112 (9.8)	20 (10.9)	11.4
Hypertensive disorders	47 (11.4)*	68 (5.9)	12 (6.5)	7.3
Infections	11 (2.7)	26 (2.3)	5 (2.7)	2.4
Intrapartum asphyxia	103 (25.0)*^	619 (54.0)	11 (62.0)	42.1
Intrauterine growth restriction	21 (5.1)*^	2 (0.2)	0 (0.0)	1.3
Maternal disease	1 (0.2)	7 (0.6)	2 (1.1)	0.6
Misc.	7 (1.7)	17 (1.5)	5 (2.7)	1.7
No obstetric cause	20 (4.9)	44 (3.8)	5 (2.7)	4.0
Spont. Preterm labour	117 (28.4)*^	190 (16.6)	18 (9.8)	18.7
Trauma	0 (0.0)	0 (0.0)	0 (0.0)	0.0
All	412	1146	184	100.0

*p < 0.05 with AGA; ^*p* < 0.05 with LGA; Pearson chi-sq tests, Fisher’s exact where cell count <5

#### Stillbirths across gestational age groups (28–32, 33–37, and 38–42 weeks)

The main causes of stillbirth at 28–32 weeks and 33–37 weeks for babies of all gestations were unexplained intrauterine death (34.5 %; 33.4 %, respectively), hypertensive disorders (27.7 %; 20.2 %) and antepartum haemorrhage (18.2 %;17.9 %). For stillbirths during the 38–42 week period the main causes were unexplained intrauterine death (33 %), intrapartum asphyxia (31.1 %) and hypertensive disorders (13.5 %) (Table 3). Mortality was highest in the 33–37 week period with death due to unexplained intrauterine death (31.5 %), hypertension (25.5 %), antepartum haemorrhage (17.0 %). Most deaths for SGA babies from spontaneous preterm labour (70.4 %), antepartum haemorrhage (64.8 %) and hypertensive disorders (57.8 %) occurred during the 33–37 week period (*p* < 0.05). Most SGA deaths from intrapartum asphyxia occurred during the 38–42 week period (*p* < 0.05).

**Table 3:** Primary cause of stillbirth for SGA babies across GA; *n* = 1042

Primary cause death	28–32w	33–37w	38–42w	All
	*n* (%)	*n* (%)	*n* (%)	*n* (%)
APH	23 (13.5)	94 (17.0)	28 (8.8)	145 (13.9)
Unexplained Intrauterine death	63 (37.1)	174 (31.5)^	130 (40.6)	367 (35.2)
Fetal abnormality	9 (5.3)	30 (5.4)	15 (4.7)	54 (5.2)
Hypertensive disorders	52 (30.6)	141 (25.5)^	51 (15.9)	244 (23.4)
Infections	3 (1.8)	9 (1.6)^	14 (4.4)	26 (2.5)
Intrapartum asphyxia	7 (4.1)	36 (6.5)^	50 (15.6)	93 (8.9)
Intrauterine growth restriction	7 (4.1)	42 (7.6)	24 (7.5)	73 (7)
Maternal disease	1 (0.6)	6 (1.1)	5 (1.6)	12 (1.2)
Spont. Preterm labour	5 (2.9)	19 (3.4)^	3 (0.9)	27 (2.6)
Trauma	0 (0.0)	1 (0.2)	0 (0)	1 (0.1)
All	170 (100.0)	552 (100.0)	320 (100.0)	1042 (100.0)

^with 38–42 week age group; Pearson chi-sq tests, Fisher’s exact where cell count <5

#### Early neonatal deaths across gestational age groups (28–32, 33–37, and 38–42 weeks)

The main causes of early neonatal death at 28–32 weeks were spontaneous preterm labour (57.4 %), hypertensive disorders (14.8 %) and antepartum haemorrhage (10.2 %). For deaths between 33 and 37 weeks the main causes of death were intrapartum asphyxia (47.3 %), fetal abnormality (17.7 %), spontaneous preterm labour (16 %) and hypertensive disorders (9.2 %). At 38–42 weeks intrapartum asphyxia was the main cause of death (77.1 %), followed by fetal abnormality (11.5 %) (Table 4). The largest causes of death during the 33–37 week period, when SGA deaths peaked, were for spontaneous preterm labour (36.5 %), fetal abnormality (18.6 %) and intrapartum asphyxia (18.6 %) (Table 4). Most deaths from intrapartum asphyxia for SGA babies occurred during the 38–42 week period (68 %), while most deaths from hypertensive disorders (48.9 %) and fetal abnormality (47 %) occurred during the 33–37 week period (*p* < 0.05).

**Table 4:** Primary cause of ENND for SGA babies at each gestation, *n* = 385

Primary cause death	28–32w	33–37w	38–42w	All
	*n* (%)	*n* (%)	*n* (%)	*n* (%)
APH	8 (9.2)	5 (3.0)	5 (3.5)	18 (1.1)
Unexplained Intrauterine death	0 (0.0)	1 (0.6)	0 (0.0)	1 (0.1)
Fetal abnormality	7 (8.0)	31 (18.6)*	28 (21.4)	66 (4.0)
Hypertensive disorders	17 (19.5)	23 (13.8)^	7 (5.3)	47 (2.9)
Infections	1 (1.1)	5 (3.0)	5 (3.8)	11 (0.7)
Intrapartum asphyxia	2 (2.3)	31 (18.6)*^	70 (53.4)	103 (6.3)
Intrauterine growth restriction	2 (2.3)	10 (6.0)	9 (6.9)	21 (1.3)
Maternal disease	1 (1.1)	0 (0.0)	0 (0.0)	1 (0.1)
Spont. Preterm labour	49 (56.3)	61 (36.5)*^	7 (5.3)	117 (7.1)
Trauma	0 (0.0)	0 (0)	0 (0)	0 (0.0)
All	87 (100.0)	167 (100.0)	131 (100.0)	385 (100.0)

*p < 0.05 with 28–32w age group; ^ with 38–42 week age group; Pearson chi-sq tests, Fisher’s exact where cell count <5

### Maternal condition

There were no significant differences in the proportion of deaths to healthy mothers by size-for-gestational-age (SGA *n* = 605, 54.9 %; AGA *n* = 1394, 55.4 %; LGA *n* = 279, 53.8 %). Of mothers who were healthy, the greatest proportion of stillbirths was seen for unexplained intrauterine death (all sizes-for-gestation 50.6 %; SGA 53.9 %). More mothers of SGA babies were hypertensive compared to AGA and LGA babies (SGA 26.3 %; AGA 21.6 %; LGA 21.2 %; *p* < 0.05), however fewer mothers of SGA babies had medical and surgical complications compared to LGA babies (SGA 5.1 %; LGA 11 %; *p* < 0.05).

Causes of stillbirth and condition of mother for SGA babies across gestational groups are presented in Table 5. Causes of early neonatal death and condition of mother for small-for-gestational age babies across gestational groups are presented in Table 6.

**Table 5:** Cause of stillbirth and condition of mother for small-for-gestational age babies, *n* = 1227

	Healthy mother			Medical surgical			Hypertension			Other maternal condition		
	28–32w	33–37w	38–42w	28–32w	33–37w	38–42w	28–32w	33–37w	38–42w	28–32w	33–37w	38–42w
Antepartum haemorrhage	10 (4.8)	20 (6.9)	8 (3.5)	2 (25.0)	4 (12.5)	0 (0.0)	3 (5.5)	16 (9.4)	4 (6.2)	19 (76.0)	62 (68.1)^	16 (42.1)
Unexplained Intrauterine death	97 (46.2)	151 (51.9)	117 (51.8)	3 (37.5)	8 (25.0)	3 (18.8)	0 (0.0)	1 (0.6)^	6 (9.2)	1 (4.0)	10 (11.0)	9 (23.7)
Fetal abnormality	21 (10.0)	24 (8.2)	14 (6.2)	0 (0.0)	3 (9.4)	0 (0.0)	2 (3.6)	7 (4.1)	1 (1.5)	0 (0.0)	1 (1.1)	0 (0.0)
Hypertensive disorders	6 (2.9)	11 (3.8)	5 (2.2)	1 (12.5)	5 (15.6)	1 (6.3)	46 (83.6)*	124 (72.9)	47 (72.3)	2 (8.0)	7 (7.7)	4 (10.5)
Infections	6 (2.9)	1 (0.3)*^	7 (3.1)	0 (0.0)	2 (6.3)	2 (12.5)	0 (0.0)	1 (0.6)	0 (0.0)	1 (4.0)	5 (5.5)	6 (15.8)
Intrapartum asphyxia	12 (5.7)	24 (8.2)	42 (18.6)	0 (0.0)	3 (9.4)	4 (25.0)	1 (1.8)	8 (4.7)	2 (3.1)	1 (4.0)	2 (2.2)	2 (5.3)
Intrauterine growth restriction	47 (22.4)	36 (12.4)*	21 (9.3)	1 (12.5)	0 (0.0)	0 (0.0)	3 (5.5)	5 (2.9)	3 (4.6)	0 (0.0)	1 (1.1)	0 (0.0)
Spont. Preterm labour	9 (4.3)	17 (5.8)^	2 (0.9)	0 (0.0)	0 (0.0)	1 (6.3)	0 (0.0)	0 (0.0)	1 (1.5)	0 (0.0)	2 (2.2)	0 (0.0)
All	210 (100)	291 (100)	226 (100)	8 (100)	32 (100)	16 (100)	55 (100)	170 (100)	65 (100)	25 (100)	91 (100)	38 (100.0)

*p < 0.05 with 28–32week group; ^*p* < 0.05 with 38–42 weeks group. Pearson’s chi-sq or Fishers exact where cell count <5

**Table 6:** Cause of Early neonatal death and condition of mother for small-for-gestational age babies, *n* = 388

	Healthy mother			Medical surgical			Hypertension			Other maternal condition		
	28–32w	33–37w	38–42w	28–32w	33–37w	38–42w	28–32w	33–37w	38–42w	28–32w	33–37w	38–42w
APH	1 (1.9)	3 (2.5)	3 (2.9)	0 (0.0)	0 (0.0)	0 (0.0)	0 (0.0)	1 (2.9)	0 (0.0)	8 (50.0)	1 (8.3)*	2 (25.0)
Unexplained Intrauterine death	0 (0.0)	1 (0.8)	0 (0.0)	0 (0.0)	0 (0.0)	0 (0.0)	0 (0.0)	0 (0.0)	0 (0.0)	0 (0.0)	0 (0.0)	0 (0.0)
Fetal abnormality	6 (11.5)	19 (16.1)	24 (23.1)	0 (0.0)	0 (0.0)	3 (50.0)	1 (5.6)	4 (11.4)	2 (13.3)	0 (0.0)	1 (8.3)	1 (12.5)
Hypertensive disorders	1 (1.9)	9 (7.6)^	1 (1.0)	0 (0.0)	0 (0.0)	1 (16.7)	16 (88.9)	21 (60.0)	5 (33.3)	0 (0.0)	1 (8.3)	0 (0.0)
Infections	0 (0.0)	3 (2.5)	3 (2.9)	0 (0.0)	0 (0.0)	1 (16.7)	0 (0.0)	0 (0.0)	0 (0.0)	1 (6.2)	2 (16.7)	2 (25.0)
Intrapartum asphyxia	2 (3.8)	24 (20.3)*	58 (55.8)	0 (0.0)	1 (33.3)	1 (16.7)	0 (0.0)	4 (11.4)^	7 (46.7)	0 (0.0)	2 (16.7)	3 (37.5)
Intrauterine growth restriction	1 (1.9)	7 (5.9)	8 (7.7)	0 (0.0)	0 (0.0)	0 (0.0)	1 (5.6)	2 (5.7)	1 (6.7)	0 (0.0)	1 (8.3)	0 (0.0)
Spont. Preterm labour	41 (78.8)	52 (44.1)	7 (6.7)	1 (100.0)	2 (66.7)	0 (0.0)	0 (0.0)	3 (8.6)	0 (0.0)	7 (43.8)	4 (33.3)	0 (0.0)
All	52 (100.0)	118 (100.0)	104 (100.0)	1 (100.0)	3 (100.0)	6 (100.0)	18 (100.0)	35 (100.0)	15 (100.0)	16 (100.0)	12 (100.0)	8 (100.0)

*p < 0.05 with 28–32week group; ^*p* < 0.05 with 38–42 weeks group. Pearson’s chi-sq or Fishers exact where cell count <5

## Discussion

Our findings indicate that there are specific characteristics unique to SGA babies in terms of gestational age at death and causes of perinatal morality.

### Timing of perinatal deaths across gestation

The same pattern of timing of stillbirth across gestation was seen for the three provinces with the greatest proportion of deaths for SGA babies during the 33–37 week period. This was in contrast to AGA and LGA babies where no peaks in the proportion of stillbirths across gestation were seen. This is consistent with observations in high-income countries where peaks have been observed between 34–37 weeks and 32–36 weeks [3, 5]. In the current study a greater proportion of ENDs for SGA babies also occurred during the 33–37 week period, suggesting that this is a critical time for both antepartum and postpartum mortality. It is also well-established that there is increased neonatal mortality for SGA preterm babies compared to AGA preterm babies [1], stressing the vulnerability of SGA babies in particular.

Current antenatal care timing differs between the provinces, with Western Cape, the most well-resourced province, continuing fortnightly antenatal care visits between 32 and 38 weeks, while Limpopo and Mpumalanga cease antenatal care visits between 32 and 38 weeks. Previous work has shown that stillbirth risk is increased during periods without antenatal care [12]. The peak in stillbirths for SGA babies seen in all provinces between 33 and 37 weeks gestation suggests that the current detection and management of SGA is not adequate even in Western Cape where frequent antenatal care visits occur.

### Causes of death/mother’s condition

Growth restriction may not necessarily play a principle role in the cause of death for all SGA babies, as SGA may also be due to slow growth of an otherwise healthy baby [4, 13]. In our study a greater proportion of SGA babies had deaths due to hypertension which is known to have placental pathology [13], indicating that growth restriction played a role in these deaths. It is important to consider that all the babies in the current cohort were considered viable (i.e. >1000 g and >28 weeks) and that very severe cases of placental insufficiency and congenital abnormalities could have died as late miscarriages. Therefore the deaths in the current study could have been potentially avoided if at risk fetuses were detected early.

Detecting SGA is complicated as in most cases the mother is healthy. In the current study there was no increase in poor maternal condition for SGA babies and more than half of all deaths had a healthy mother. Of mothers that were healthy the greatest proportion of SGA stillbirths were due to unexplained intrauterine death. Earlier detection of fetuses at risk antenatally may reduce the number of unexplained intrauterine deaths.

### Challenges with detection

Better detection of SGA babies is needed in South Africa and LMICs. Palpation and symphysis fundal height are commonly used in LMICs due to limited alternative resources, despite the limited evidence to support this as an effective method to predict growth restriction [14]. Clinical trials in high-income countries estimate that up to 76 % of SGA cases can be detected antenatally [15]. The use of Doppler velocimetry to measure altered umbilical artery blood flow in high risk women has enhanced the ability to detect fetuses with pathological growth restriction [16], reducing perinatal mortality [17]. However, challenges with Doppler measurement in South Africa are present as there are a large number of perinatal deaths to low-risk healthy mothers [7] and who would therefore not be referred for screening. Studies in South Africa have explored the use of a continuous wave Doppler analyser using a PC (Umbiflow), a simpler alternative to umbilical artery Doppler, which can be operated by nurses and midwives at the primary health care centre level [18, 19]. The Umbiflow is able to detect fetuses at risk of stillbirth based on abnormal umbilical artery blood flow. While meta-analyses in high-income countries found Doppler use in low-risk pregnancies to be ineffective to reduce perinatal mortality and morbidity [20], there may be potential benefit of Doppler or Umbiflow use in low risk populations in LMICs where stillbirth rates are higher. However, once a fetus at risk of stillbirth is detected, quality clinical care must also be provided to increase the risk of survival.

### Challenges with clinical management

Once SGA has been detected there are challenges in the clinical management of such babies. Currently there are no effective approaches for the reversal or improvement of the growth pattern of a fetus [21], therefore prenatal clinical management is focused on identifying the optimal timing of delivery. The gestational age of the fetus is a critical component of the decision-making process, as fetal mortality is lower than neonatal mortality prior to 31 weeks [22], and delivery after 39 weeks results in increased perinatal mortality [23]. While it is known that the neonatal mortality risk is higher for preterm SGA babies compared to term SGA babies [1], it unclear how much size-for-gestational age impacts the risk of neonatal mortality in the preterm compared to late preterm infant [24, 25]. It has recently been suggested that the delivery of SGA babies around 37 weeks is optimal to avoid increased risk of stillbirth occurring after week 37 [26]. Causes of death for SGA babies in other studies have shown different patterns across gestation with a greater proportion of deaths due to congenital abnormality (32–42 weeks) and intrapartum asphyxia (37–42 weeks) at older gestations [5]. We observed similar findings in our data with SGA deaths from intrapartum asphyxia and fetal abnormality occurring more frequently at older gestations. Standard guidelines for managing labour in South African include partogram and monitoring of fetal heart rate with a fetal stethoscope or doptone in Community Health Centres and electronically at district hospitals and above. However it is estimated that in 44 % of deaths these guidelines are not adhered to due to limited resources and staff [27]. Caesarean delivery is not readily accessible in some districts, and long transport times exist between Community Health Centres to facilities that are able to perform caesarean section deliveries. It is likely these factors play a role in the high proportion of deaths due to fetal asphyxia observed in the current study. It is also important to note that spontaneous preterm labour is an underlying cause of early neonatal death where the maternal condition contributes to increased risk of death from disorders associated with prematurity. Early neonatal deaths for preterm neonates reflect access and quality of care after birth for preterm infants. The final cause of death for a preterm neonate could be due to a causes such as hyaline membrane disease, hypoxic ischaemic encephalopathy and meconium aspiration. Doppler measurements in combination with growth charts can be used to inform the management of SGA pregnancies [17]. This has been shown to decrease the induction of labour and hospital admissions in high-income countries [17].

### Limitations

There are some limitations to the current study. First, there is likely some ambiguity in the assignment of gestational age from the perinatal audit data. Last menstrual period or ultrasound estimates for gestational age may not be accurate and the SGA category may erroneously capture appropriately sized infants who measure small for their misassigned gestational age. We sought to reduce this issue by using only data where gestational age estimates were considered ‘certain’. Second, macerated SGA babies are likely to be overestimated as death may have occurred up to 2 weeks prior [28] thus the fetus may have been appropriate-for-gestational age at the time of death. Third, as we had access to aggregated data at a centre level, and data for the number of live births for SGA, AGA and LGA were unavailable we were unable to calculate stillbirth risk, relative risks between live and stillborn babies or perform individual unit analysis using multivariable regression. Fourth, both multiple and single gestations were included in the current study. These were not separated due to limited statistical power and therefore any differences in patterns of mortality between multiple and single gestations are not presented in the current study. Deaths directly related to multiple gestation such as twin-to-twin transfusion only represented 0.3 % of all deaths in the current study, therefore it is unlikely that the inclusion of multiple gestations introduced significant bias.

## Conclusion

Mortality from SGA in South Africa accounts for a considerable number of deaths, yet there is little research on the timing, causes and detection of SGA in low-resource settings. The detection and management of SGA is important as most deaths occur in late preterm or term infants, where the chance of survival is high when adequate care can be delivered. If SGA infants can be detected antenatally and managed effectively there is an opportunity to significantly reduce the burden of perinatal mortality in LMICs. Further studies on the relationship between antenatal detection and outcome of SGA are needed, especially in LMICs.

## Abbreviations

AGA: Appropriate-for-gestational-age
END: Early neonatal death (up to 7 days neonatal life)
IUGR: Intrauterine growth restriction
LGA: Large-for-gestational-age
LMIC: Low-and-middle-income-countries
PPIP: Perinatal Problems Identification Program
SGA: Small-for-gestational-age
